# Risk factors for avascular necrosis in patients with systemic lupus erythematosus: a multi-center cohort study of Chinese SLE Treatment and Research Group (CSTAR) Registry XXII

**DOI:** 10.1186/s13075-023-03061-3

**Published:** 2023-05-12

**Authors:** Cheng Cheng, Can Huang, Zhen Chen, Feng Zhan, Xinwang Duan, Yongfu Wang, Cheng Zhao, Zhenbiao Wu, Jian Xu, Hongbin Li, Min Yang, Rui Wu, Jiuliang Zhao, Shangzhu Zhang, Qian Wang, Xiaomei Leng, Xinping Tian, Mengtao Li, Xiaofeng Zeng

**Affiliations:** 1 Department of Rheumatology and Clinical Immunology, Peking Union Medical College Hospital, Chinese Academy of Medical Sciences, Peking Union Medical College, National Clinical Research Center for Dermatologic and Immunologic Diseases (NCRC-DID), Ministry of Science & Technology, State Key Laboratory of Complex Severe and Rare Diseases, Peking Union Medical College Hospital, Key Laboratory of Rheumatology and Clinical Immunology, Ministry of Education, Beijing, 100730 China; 2 Department of Rheumatology and Immunology, The Second Affiliated Hospital of Fujian Medical University, Quanzhou, Fujian China; 3 Department of Rheumatology, Hainan General Hospital, Haikou, China; 4 Department of Rheumatology, The Second Affiliated Hospital of Nanchang University, Nanchang, China; 5 Department of Rheumatology Institute of Immunology and Rheumatology Inner Mongolia Key Laboratory of Autoimmunity, The First Affiliated Hospital of Baotou Medical College, Baotou Medical College, Baotou, China; 6 Department of Rheumatology and Clinical Immunology, The First Affiliated Hospital of Guangxi Medical University, Nanning, China; 7 Department of Clinical Immunology and Rheumatology, Xijing Hospital Affiliated to the Fourth Military Medical University, Xi’an, China; 8 Department of Rheumatology, First Affiliated Hospital of Kunming Medical University, Kunming, China; 9 Department of Rheumatology, Affiliated Hospital of Inner Mongolia Medical College, Hohhot, China; 10 Department of Rheumatology and Immunology, Nanfang Hospital, Southern Medical University, Guangzhou, China; 11 Department of Rheumatology, The First Affiliated Hospital of Nanchang University, Nanchang, China

**Keywords:** Systemic lupus erythematosus, Avascular necrosis, Risk factors, Risk stratification

## Abstract

**Background:**

Avascular necrosis is a common organ damage in SLE patients, which can influence patients’ life quality. Conflicting results exist in risk factors of AVN in SLE patients. The aim of this study was to illustrate risk factors predicting the occurrence of avascular necrosis (AVN), also known as osteonecrosis, in systemic lupus erythematosus (SLE) patients in Chinese SLE Treatment and Research Group (CSTAR), a multi-center cohort of Chinese SLE patients.

**Methods:**

SLE patients in CSTAR without existing AVN at registration were included. At least two follow-ups and an observation period of no less than 2 years for AVN event were required. Univariate and multivariate Cox regression analyses were used to evaluate risk factors for AVN in SLE patients. Coefficient B was transformed to risk score for the development of a risk stratification model.

**Results:**

One hundred six (2.59%) of 4091 SLE patients were diagnosed AVN during follow-ups of no less than 2 years. Multi-variate Cox regression analysis suggested that SLE onset age ≤ 30 (HR 1.616, *p* 0.023), arthritis (HR 1.642, *p* 0.018), existing organ damage (SDI ≥ 1) at registration (HR 2.610, *p* < 0.001), positive anti-RNP (HR 1.709, *p* 0.006), and high glucocorticoid maximum daily dose at registration (HR 1.747, *p* 0.02) were independent risk factors. A risk stratification system was developed according to the risk factors, and patients were divided into high risk (3–6) and low risk (0–2). The AUC of 0.692 indicated moderate discrimination. The calibration curve in internal validation was drawn.

**Conclusion:**

Patients with SLE onset age ≤ 30, arthritis, existing organ damage (SDI ≥ 1) at registration, positive anti-RNP, and high glucocorticoid maximum daily dose at registration are at high risk for AVN and require attention.

## Introduction

Systemic lupus erythematosus (SLE) is an autoimmune disease involving multiple organs/systems [1]. Organ damages can occur during disease course and cause irreversible consequences on patients’ health and life quality [2]. It was reported that the incidence rate of avascular necrosis (AVN) in SLE was 3–40% [3, 4]. More than one site can be affected in AVN, including unilateral or bilateral femoral head, knee joint, and so on [3]. AVN commonly begins with obscure symptoms and can progress to severe pain and movement restriction, requiring arthroplasty in the late stage. Therefore, evaluating risk factors and developing a risk stratification system for AVN in SLE patients is crucial for clinicians to recognize patients with higher risk for AVN and to facilitate personalized clinical decisions for disease monitoring and early interventions.

The mechanisms of AVN development in SLE patients are complicated and not fully elucidated. Glucocorticoid (GC) usage has been revealed as a crucial risk factor in various researches [5, 6]. However, studies also indicated that SLE patients obtained even higher risks for AVN when compared with other patients administered glucocorticoids [7], and major organ involvement in SLE might play an important role in the occurrence of AVN [8]. It has been reported that Asian patients have even higher risks for AVN than non-Asian patients [9].

However, to our knowledge, no multi-center cohort study has been conducted to explore risk factors for AVN in Chinese SLE patients. The aim of this study was to evaluate risk factors for AVN in SLE patients registered in Chinese SLE Treatment and Research Group (CSTAR), and to arouse attention to high-risk patients for early detection and intervention.

## Methods

### Patients

This study was based on data from CSTAR, a multi-center Chinese SLE cohort, in which 331 rheumatology centers nationwide participate. The inclusion criteria were fulfillment of the 1997 SLE classification criteria revised by the American College of Rheumatology (ACR) [10] or 2012 Systemic Lupus International Collaborating Clinics (SLICC) classification criteria for SLE [2]. 4091 SLE patients in CSTAR who fulfilled the criteria of SLE classification without existing AVN at registration and underwent at least 2 follow-ups with no less than 2 years of observation period for AVN were included. Patients included were registered between February 2009 and Jan 2021. Patients were excluded if they were diagnosed AVN before registration. The study has been approved by the Medical Ethics Committee of the leading site, Peking Union Medical College Hospital (PUMCH) (IRB number: S-478). Other centers were also approved by their ethics committee if locally required. All patients have signed written informed consent before registration.

### Data collection

Uniform protocol was used in all centers of CSTAR for data acquisition and evaluation [11]. At the time of registration, basic information including gender, date of birth, time of SLE onset, and diagnosis were recorded. Also, organ/system involvement was documented at the time of registration and updated in follow-ups, including mucocutaneous, renal, neuropsychiatric, hematological involvement, serositis, arthritis, and pulmonary arterial hypertension. Autoantibody profiles were documented, including antinuclear antibody (ANA), anti-double stranded DNA antibody (anti-dsDNA), anti-Smith antibody (anti-Sm), anti-ribonucleoprotein antibody (anti-RNP), anti-Sjogren Syndrome A antibody (anti-SSA), anti-Sjogren Syndrome B antibody (anti-SSB), anti-ribosomal P protein antibody (anti-rib P), anticardiolipin antibody (aCL), anti-β2 glycoprotein 1 antibody (anti-β2GP1), and lupus anticoagulant (LA). Other laboratory exams including Coombs test and hypocomplementemia were also evaluated. Systemic Lupus Erythematosus Disease Activity Index 2000 (SLEDAI-2K) was used to define patients’ disease activity state. Information about organ damages according to the SLICC/ACR Damage Index (SDI) [2] was obtained from physician-recorded data at follow-ups or patient self-reported electronic or telephone questionnaires. Treatment strategies were also documented at registration, and a daily dose of GC prednisone equivalent ≥60mg was defined as high dose GC.

### Identification of AVN

Time of AVN was defined when patients were firstly diagnosed by medical institutions as AVN, with/without symptoms of joint pain and movement restriction, mostly having radiographic evidence including magnetic resonance imaging (MRI), computed tomography, or X-ray. The event of AVN was recorded by clinicians during follow-ups, or by patient self-reported electronic or telephone questionnaires.

### Statistical analysis

Categorical data were shown as percentages. Continuous data in common distribution were displayed in mean and standardized error (SE) and continuous data not in common distribution were displayed in median and interquartile range (IQR). Student’s *t* test was used for comparing continuous variables in common distribution. Mann-Whitney *U* test was used to test significance for discrete variables or continuous variables not in common distribution. Pearson chi-squared test or Fisher’s exact test was used for categorical variables. Univariate and multivariate Cox regression analyses were employed to evaluate predicting factors for AVN in SLE patients. *P*<0.05 was considered statistically significant. The statistical analysis was performed by SPSS 21.0 and R software (3.6.1).

### Development of risk stratification system

A risk scoring system was established based on potential risk factors for AVN found in multivariate Cox regression analysis. The regression coefficient *B* was used to develop risk score. The score of each risk factor was calculated as |B/Bmin|. The risk score was calculated for each patient. Receiver operating characteristic curve (ROC curve) was drawn and area under the receiver operating characteristic curve (AUC) was calculated to estimate the best cut-off point. The internal validation was shown with calibration curve.

## Results

### Characteristics of SLE patients with and without AVN upon registration

**Fig. 1:**
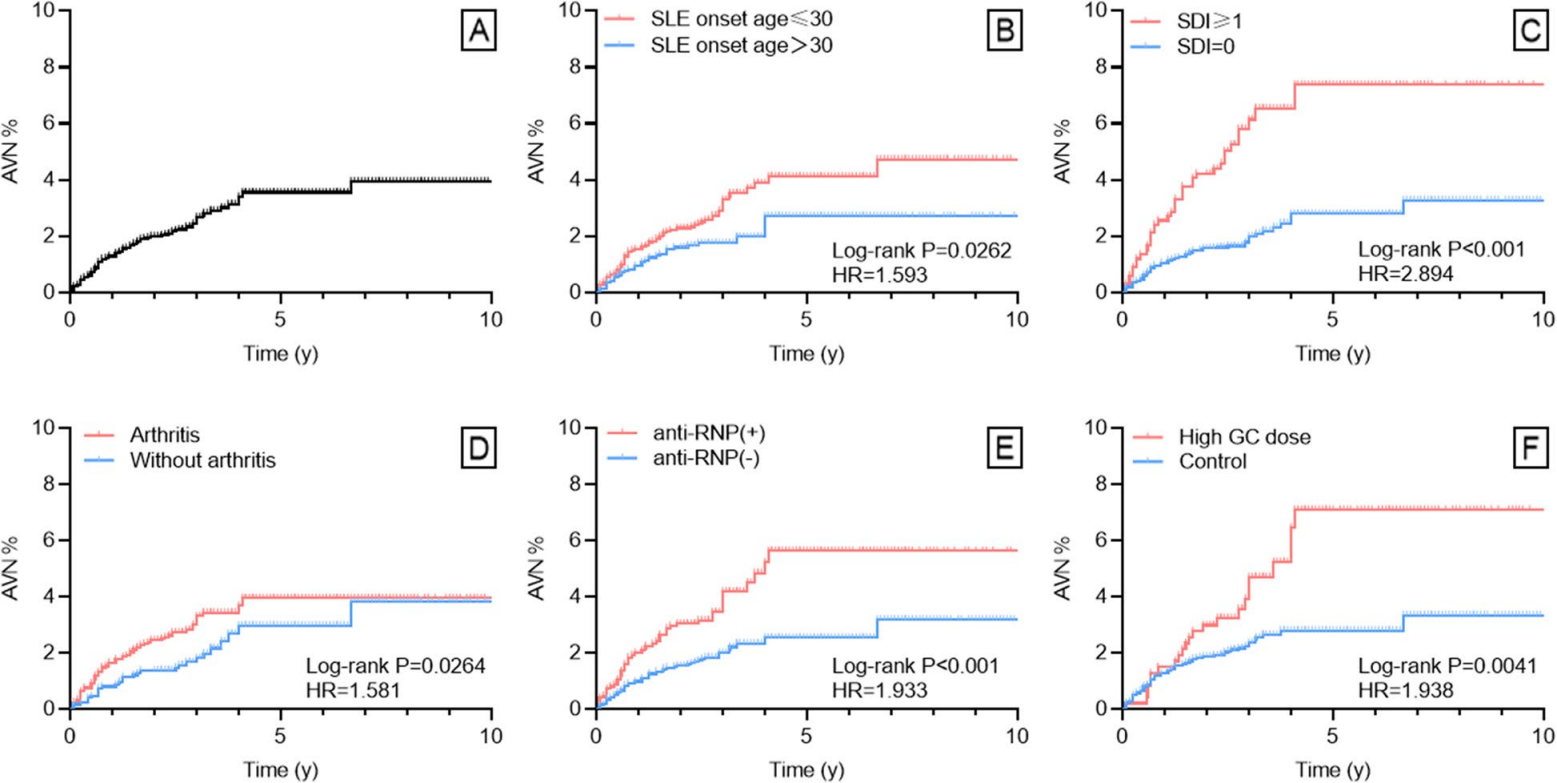
Kaplan–Meier curves for the percentage of avascular necrosis (AVN) occurrence in systemic lupus erythematosus (SLE) patients

A total of 106 patients developed AVN after registration during follow-ups of no less than 2 years. The median age at the identification of AVN was 31.00 years old (IQR 27.19, 40.27). The time interval from SLE diagnosis to AVN was 3.76 years (1.75, 6.50). As was shown in Table 1, 97.2% (103) SLE-AVN patients have started the usage of GC up until registration. The median time from the initiation of GC to the development of AVN was 1.37 years (0.56–2.86). Figure 1A illustrated the percentage of AVN development in SLE patients over time. Table 2 displayed the characteristics of AVN in SLE patients. Among the 106 SLE-AVN patients, 87 reported single/multiple lesion sites, and 58 (82.1%) had more than one site involved. Among the 33 patients who reported more specific information about AVN, femoral head was the most common AVN site with prevalence of 87.8% and mostly bilaterally involved, while 27.3% had unilateral or bilateral knee joints affected. Most patients were diagnosed AVN via MRI (69.7%). As for consequences, 18.2% patients underwent surgery of femoral head replacement.

**Table 1:** Demographic, clinical, and laboratory characteristics of SLE patients with and without AVN

	**All (*n* = 4091)**	**SLE-AVN (*n* = 106)**	**SLE-non-AVN (*n* = 3985)**	***p* value**
Demographic and clinical characteristics				
Female,* N* (%)	3848 (94.1%)	98 (92.5%)	3750 (94.1%)	0.478
Age at SLE onset (y), median (25%, 75%IQR)	27.51 (21.15, 36.15)	25.76 (21.38, 30.81)	27.58 (21.15, 36.26)	0.060
Disease duration at registration (y), median (25%, 75%IQR)	2.09 (0.17, 5.92)	2.01 (0.42, 5.58)	2.09 (0.17, 5.92)	0.919
SLEDAI-2 K, median (25%, 75%IQR)	3 (0, 8)	2 (0, 7)	3 (0, 8)	0.318
Existing organ damage (SDI ≥ 1) at registration, *N* (%)	663 (16.2%)	38 (35.8%)	625 (15.7%)	< 0.001**
Cataract, *N* (%)	23 (0.6%)	3 (2.8%)	20 (0.5%)	0.021*
PAH, *N* (%)	191 (4.7%)	16 (15.1%)	175 (4.4%)	< 0.001**
Osteoporosis with fracture or vertebral collapse, *N* (%)	14 (0.3%)	2 (1.9%)	12 (0.3%)	0.049*
Premature gonadal failure, *N* (%)	24 (0.6%)	3 (2.8%)	21 (0.5%)	0.023*
Mucocutaneous, *N* (%)	3271 (80.0%)	89 (84.0%)	3182 (79.8%)	0.297
Renal, *N* (%)	1598 (39.1%)	38 (35.8%)	1560 (39.1%)	0.492
Neuropsychiatric, *N* (%)	362 (8.8%)	15 (14.2%)	347 (8.7%)	0.051
Hematological, *N* (%)	1904 (46.5%)	60 (56.6%)	1844 (46.3%)	0.035*
Serositis, *N* (%)	460 (11.2%)	20 (18.9%)	440 (11.0%)	0.012*
Arthritis, *N* (%)	2359 (57.7%)	72 (67.9%)	2287 (57.4%)	0.030*
PAH, *N* (%)	203 (5.0%)	16 (15.1%)	187 (4.7%)	< 0.001**
Laboratory results up until registration				
Hypocomplementemia,* N* (%)	2934 (71.7%)	82 (77.4%)	2852 (71.6%)	0.191
ANA, *N* (%)	4006 (97.9%)	104 (98.1%)	3902 (97.9%)	1.000
Anti-dsDNA, *N* (%)	3138 (76.7%)	81 (76.4%)	3057 (76.7%)	0.943
Anti-Sm, *N* (%)	3092 (75.6%)	52 (49.1%)	3007 (75.5%)	0.263
Anti-RNP, *N* (%)	1247 (30.5%)	49 (46.2%)	1198 (30.1%)	< 0.001**
Anti-SSA, *N* (%)	1906 (46.6%)	53 (50.0%)	1853 (46.5%)	0.476
Anti-SSB, *N* (%)	673 (16.5%)	18 (17.0%)	655 (16.4%)	0.881
Anti-rib P, *N* (%)	642 (15.7%)	25 (23.6%)	617 (15.5%)	0.024*
Positivity in aPLs, *N* (%)	695 (17.0%)	20 (18.9%)	675 (16.9%)	0.602
Coombs, *N* (%)	2188 (53.5%)	52 (49.1%)	2136 (53.6%)	0.355
GC usage, *N* (%)	3505 (85.7%)	103 (97.2%)	3402 (85.4%)	0.001**
GC maximum dose at registration(mg), median (25%, 75%IQR)	10.0 (5.0, 35.0)	13.8 (10.0, 50.0)	10.0 (3.8, 30.0)	< 0.001**
High dose GC at registration, *N* (%)	468 (11.4%)	23 (21.7%)	445 (11.2%)	0.001**
Hydroxychloroquine, *N* (%)	3199 (78.2%)	76 (71.7%)	3123 (78.4%)	0.101
Immunosuppressants, *N* (%)	2561 (62.6%)	73 (68.9%)	2488 (62.4%)	0.177

aPLs include anti-β2GP1 antibody, anti-cardiolipin antibody, and lupus anticoagulant

*SLE* systemic lupus erythematosus, *AVN* avascular necrosis, *SLEDAI-2 K* Systemic Lupus Erythematosus Disease Activity Index 2000, *PAH* pulmonary arterial hypertension, *SDI* Systemic Lupus International Collaborating Clinics/American College of Rheumatology Damage Index, *GC* glucocorticoid

*p < 0.05, **p < 0.01

**Table 2:** Characteristics of AVN in SLE patients

Age at AVN, Median (25%, 75%IQR)	31.00 (27.19, 40.27)
Multiple locations, *N* (%)	58^a^ (82.1%)
Locations of AVN, *N* (%)	
Femoral head, *N* (%)	29^b^ (87.8%)
Bilateral femoral heads, *N* (%)	20^b^ (60.6%)
Knee joint, *N* (%)	9^b^ (27.3%)
Bilateral knee joints, *N* (%)	4^b^ (12.1%)
Diagnostic tools	
MRI, *N* (%)	23^b^ (69.7%)
CT only, *N* (%)	2^b^ (6.1%)
X-ray only, *N* (%)	5^b^ (15.2%)
Femoral head replacement, *N* (%)	6^b^ (18.2%)

^a^Among 106 SLE-AVN patients, 87 reported single or multiple lesion sites in this study

^b^Among 106 SLE-AVN patients, 33 patients reported specific lesions involved, diagnostic tools, and surgeries performed

The clinical manifestations up until registration in the SLE-AVN group (*N*=106) and the SLE-non-AVN group (*N*=3985) were compared (Table 1). Patients in the SLE-AVN group showed significantly higher prevalence of hematological involvement (SLE-AVN 56.6%, SLE-non AVN 46.3%, *p*=0.035), serositis (SLE-AVN 18.9%, SLE-non AVN 11.0%, *p*=0.012), arthritis (SLE-AVN 67.9%, SLE-non AVN 57.4%, *p*=0.03), and pulmonary arterial hypertension (PAH) (SLE-AVN 15.1%, SLE-non AVN 4.7%, *p*<0.001). SLE-AVN patients (38, 35.8%) obtained significantly higher rates of existing organ damage (SDI≥1) than the SLE-non AVN group (625, 15.7%) (*p*<0.001) at registration, among which the prevalence of cataract, PAH, osteoporosis with fracture or vertebral collapse, and premature gonadal failure were significantly higher in the SLE-AVN group than the non-AVN group. No significant difference in SLEDAI-2K at registration was observed.

The immunological profiles of the SLE-AVN and SLE-non AVN groups were also demonstrated in Table 1. Up until registration, a higher rate of positive anti-RNP and anti-rib P in the SLE-AVN group was revealed (*p*<0.05).

The treatment strategies were also illustrated (Table 1). At registration, 3505 (85.7%) patients started glucocorticoid administration, 3199 (78.2%) patients initiated the use of hydroxychloroquine, and 2561 (62.6%) used other immunosuppressants. The rate of glucocorticoid administration was significantly higher in the SLE-AVN group. Additionally, the maximum daily dose of glucocorticoid was significantly higher in the SLE-AVN group than in the SLE-non AVN group.

### Risk factors for AVN in SLE patients

Univariate Cox analysis was used to evaluate potential risk factors for AVN development in SLE patients (Table 3). Clinical factors with statistical significance included age at SLE onset ≤30 (HR 1.594, 95%CI 1.052–2.413, *p* =0.028), serositis (HR 1.706, 1.048–2.779, *p* =0.032), arthritis (HR 1.581, 1.051–2.377, *p* =0.028), PAH (HR 3.064, 1.797–5.227, *p* <0.001), and existing organ damage (SDI≥1) at registration (HR 2.894, 1.946–4.305, *p* <0.001). The presence of positive anti-RNP (HR 1.934, 1.320–2.834, *p*=0.001) and anti-rib P antibodies (HR 1.630, 1.040–2.552, *p*=0.033), and high dose GC maximum daily dose at registration (HR 1.949, 1.225–3.100, *p* =0.005) were also risk factors in univariate Cox analysis (Table 3).

**Table 3:** Univariate and multivariate Cox regression analyses of risk factors of AVN in SLE patients

	**Univariate Cox analysis**		**Multivariate Cox analysis**	
	**HR (95% CI)**	***p* value**	**HR (95% CI)**	***p* value**
Demographic characteristics				
Gender	0.747 (0.363–1.536)	0.428		
Age at SLE onset ≤ 30	1.594 (1.052–2.413)	0.028*	1.616 (1.067–2.449)	0.023*
SLEDAI-2 K	0.983 (0.950–1.017)	0.317		
Existing organ damage (SDI ≥ 1) at registration	2.894 (1.946–4.305)	< 0.001**	2.610 (1.748–3.895)	< 0.001**
Organ/system involvement up until registration				
Mucocutaneous	1.332 (0.793–2.238)	0.278		
Renal	0.845 (0.568–1.258)	0.407		
Neuropsychiatric	1.715 (0.993–2.962)	0.053		
Hematological	1.445 (0.984–2.123)	0.061		
Serositis	1.706 (1.048–2.779)	0.032*		
Arthritis	1.581 (1.051–2.377)	0.028*	1.642 (1.089–2.475)	0.018*
PAH	3.064 (1.797–5.227)	< 0.001**		
Laboratory results up until registration				
Hypocomplementemia	1.305 (0.828–2.058)	0.251		
ANA	0.978 (0.241–3.965)	0.975		
Anti-dsDNA	1.008 (0.643–1.578)	0.973		
Anti-Sm	1.393 (0.863–2.247)	0.175		
Anti-RNP	1.934 (1.320–2.834)	0.001**	1.709 (1.162–2.515)	0.006**
Anti-SSA	1.110 (0.758–1.625)	0.592		
Anti-SSB	1.023 (0.616–1.699)	0.929		
Anti-rib P	1.630 (1.040–2.552)	0.033*		
Positivity in aPLs	1.180 (0.726–1.921)	0.504		
Coombs	0.958 (0.651–1.409)	0.827		
Treatment up until registration				
High dose GC at registration	1.949 (1.225–3.100)	0.005**	1.747 (1.092–2.795)	0.020*
Hydroxychloroquine	0.706 (0.463–1.077)	0.106		
Immunosuppressants	1.332 (0.883–2.009)	0.172		

Antiphospholipid antibodies (aPLs) include anti-β2GP1 antibody, anti-cardiolipin antibody (aCL), and lupus anticoagulant (LA)

*SLE* systemic lupus erythematosus, *AVN* avascular necrosis, *SLEDAI-2 K* Systemic Lupus Erythematosus Disease Activity Index 2000, *PAH* pulmonary arterial hypertension, *SDI* Systemic Lupus International Collaborating Clinics/American College of Rheumatology Damage Index, *GC* glucocorticoid

*p < 0.05, **p < 0.01

**Fig. 2:**
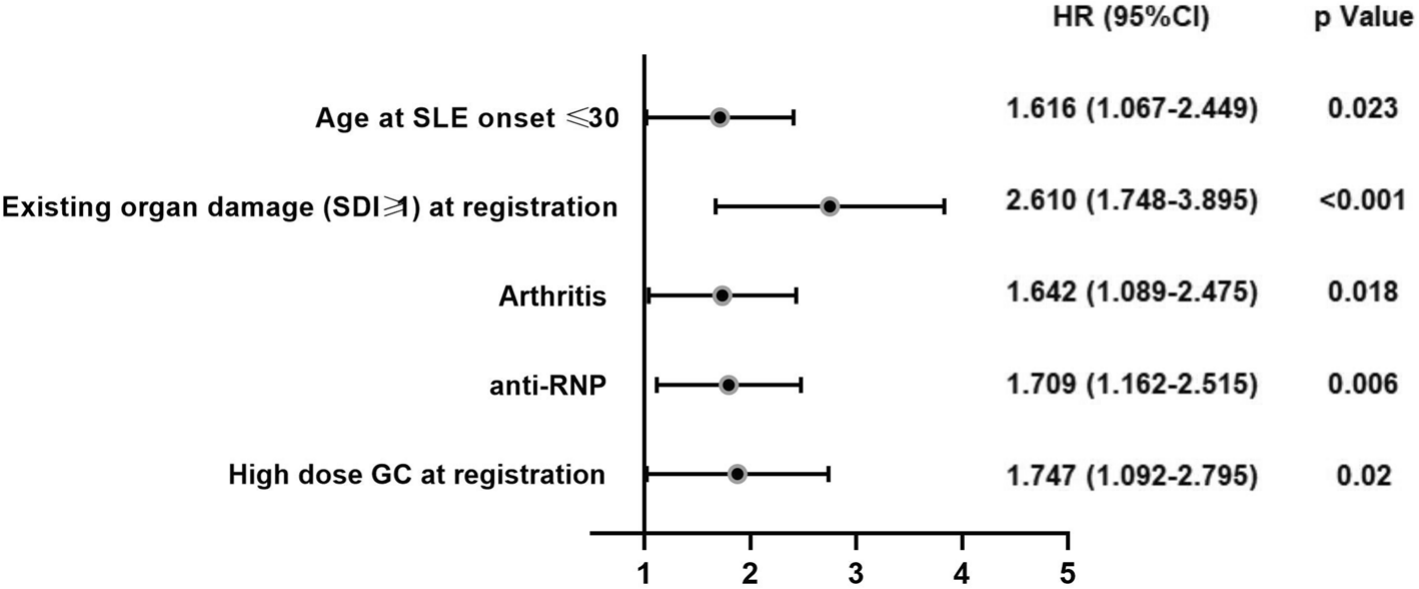
Forest plot of the risk factors in multivariate Cox regression analysis

Multivariate Cox regression analysis was performed to include variables with *p* < 0.05 in univariate Cox analysis and variables considered clinically important. Variables included in multivariate Cox analysis were SLE onset age≤30, serositis, arthritis, PAH, anti-RNP positivity, anti-rib P positivity, existing organ damage (SDI≥1) at registration, and high GC maximum daily dose at registration. As shown in Table 3 and Fig. 2, SLE onset age≤30 (HR 1.616, 95%CI 1.067–2.449, *p*=0.023), arthritis (HR 1.642, 95%CI 1.089–2.475, *p*=0.018), existing organ damage (SDI≥1) at registration (HR 2.610, 95%CI 1.748–3.895, *p*<0.001), positive anti-RNP (HR 1.709, 95%CI 1.162–2.515, *p*=0.006), and high glucocorticoid maximum daily dose at registration (HR 1.747, 95%CI 1.092–2.795, *p*=0.02) were independent risk factors for AVN development. And the percentages of AVN occurrence in SLE patients with and without these risk factors were shown in Fig. 1B–F.

### The prediction model of AVN and internal validation

**Fig. 3:**
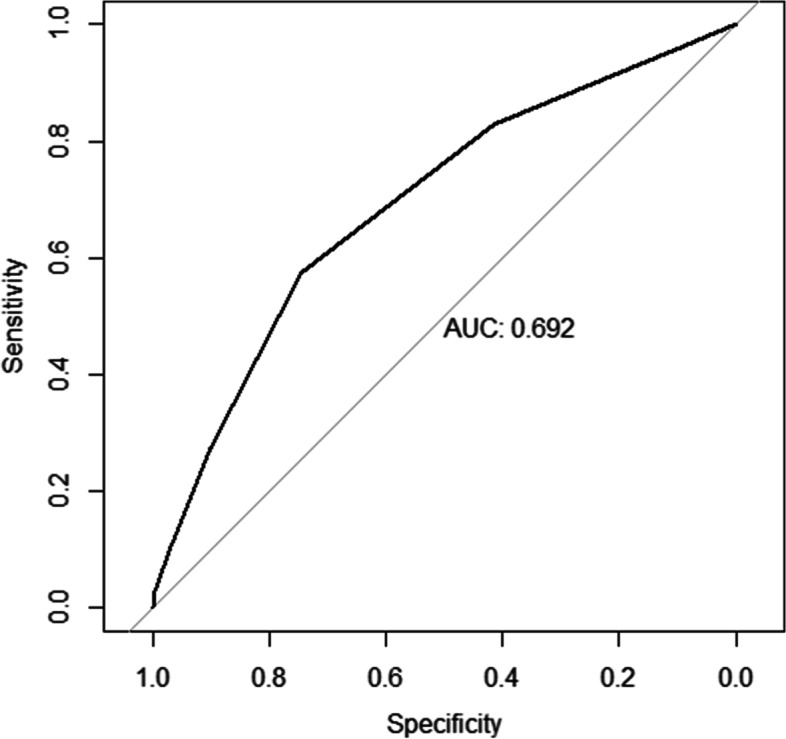
ROC curve of the predictive model

**Fig. 4:**
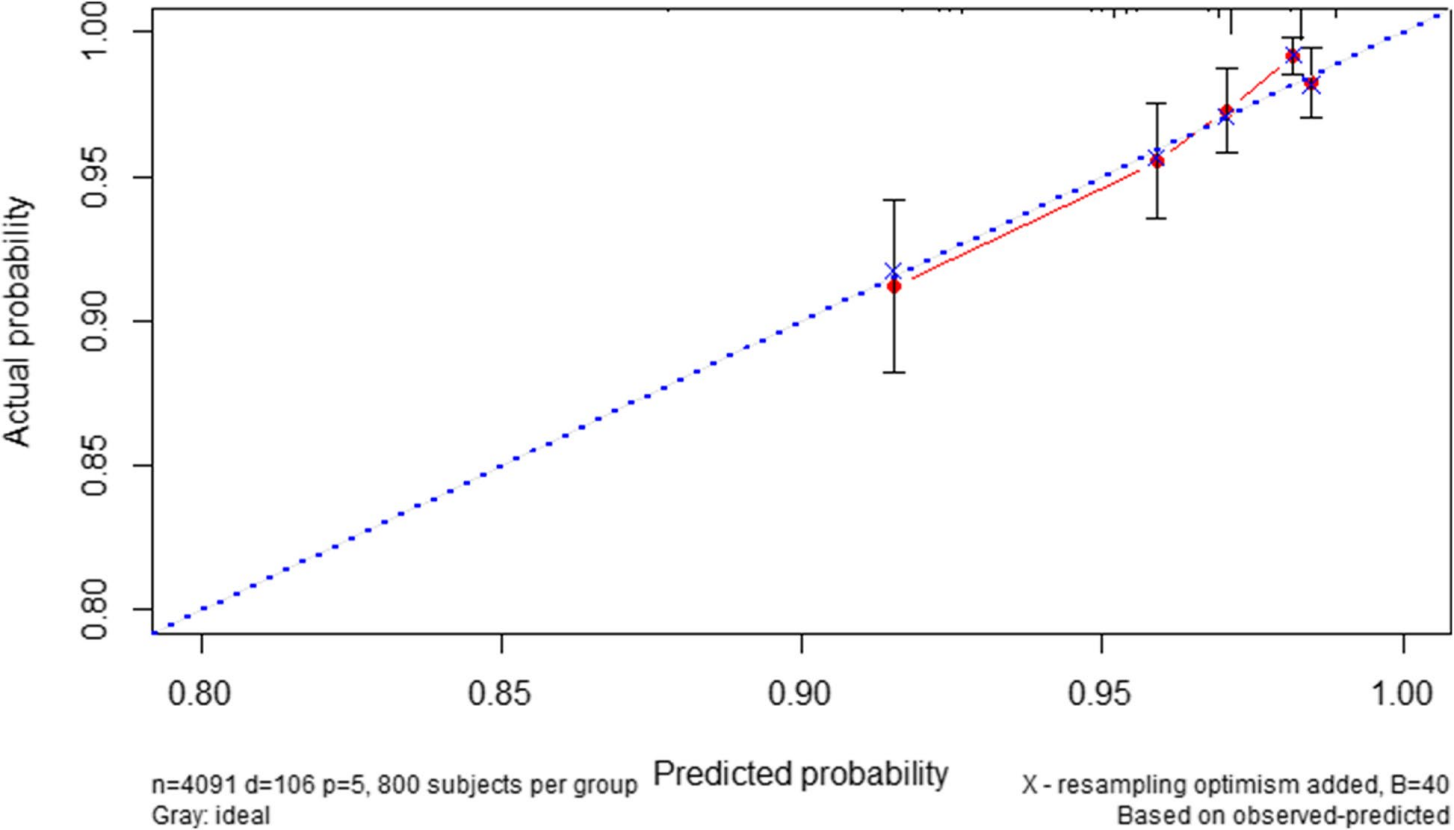
The calibration curve in internal validation

The hazard ratio and B regression coefficient in the multivariate Cox analysis model were demonstrated in Table 4. The scores of age at SLE onset ≤ 30, existing organ damage (SDI ≥ 1) at registration, arthritis, anti-RNP positivity, and high GC maximum dose at registration were 1, 2, 1, 1, 1 points respectively. We calculated the risk score for each SLE patient and drawn the ROC curve of the predictive model (Fig. 3). A score of 2 was the best cut-off point with sensitivity of 0.746 and specificity of 0.549. The risk stratification for AVN according to the risk scores was shown in Table 5. The risk scores were divided into high risk (3–6) and low risk (0–2), and the risks for AVN were 5.7% and 1.5% respectively. The AUC of 0.692 indicated that the model had moderate discrimination. The calibration curve in internal validation was drawn in Fig. 4.

**Table 4:** Hazard ratio and B coefficient with multivariable Cox regression model and corresponding risk score

**Variables**	**Hazard ratio**	***B* coefficient**	**Score**
Age at SLE onset ≤ 30	1.616 (1.067–2.449)	0.480	1
Existing organ damage (SDI≥1) at registration	2.610 (1.748–3.895)	0.959	2
Arthritis	1.642 (1.089–2.475)	0.496	1
Anti-RNP	1.709 (1.162–2.515)	0.536	1
High dose GC at registration	1.747 (1.092**–**2.795)	0.558	1

*SLE* systemic lupus erythematosus, *GC* glucocorticoid

**Table 5:** The risk stratification for AVN according to the risk scoring system

**Risk stratification group**	**Number of patients**	**AVN, *N* (%)**
Low risk (0–2)	3019	45(1.5%)
High risk (3–6)	1072	61 (5.7%)
Total	4091	106 (2.6%)

## Discussion

This study demonstrated the clinical characteristics of SLE-AVN and predicting factors for AVN development based on analysis of patients registered in CSTAR. Among the 4091 patients included, 106 patients (2.59%) developed AVN in an observation period of no less than 2 years after registration. Multivariate Cox regression analysis revealed independent risk factors for AVN were age at SLE onset≤30, arthritis, positive anti-RNP antibody, existing organ damage (SDI≥1) at registration, and higher maximum daily dose of GC at registration, which were also included in the risk scoring predictive model to discriminate SLE patients with higher risk of AVN from those with lower risk.

Patients developing symptoms of SLE at the age ≤30 were at a higher risk of AVN in this study, which was in accordance with a previous study showing that the mean age of SLE onset was younger in SLE-AVN patients than SLE-non-AVN patients [12]. In another study, the prevalence of AVN in SLE patients peaked among those aged 20–29 years, while none of the patients younger than 14 years developed AVN [13]. However, it remained unclear why AVN developed more frequently in younger patients compared with older ones among adult SLE patients. Further researches with more clinical data and larger samples are needed to confirm the current findings.

Various studies have indicated GC as a crucial risk factor for AVN in SLE patients [14], especially higher dose GC [6, 7, 15, 16]. Glucocorticoids can directly injure endothelial cells, enhance hypercoagulability, induce fat cell hypertrophy, and inhibit angiogenesis, which may result in reduced blood flow and oxygen delivery through micro-vessels, leading to bone ischemia and an increased risk of developing AVN [17]. Our study revealed high GC maximum daily dose (prednisone equivalent ≥60mg/d) as a risk factor for AVN development. Nakamura et al. found that delayed osteonecrosis occurred only in patients with SLE recurrence requiring increased glucocorticoid doses [18]. The results of the aforementioned studies supported that osteonecrosis was associated with high-dose corticosteroid treatment. It has been revealed in a retrospective study that disease duration of more than 5 years and cumulative use of GC were significant predictors for AVN in SLE patients in univariate logistic regression analysis, while a disease duration of more than 5 years was an independent predictor in multivariate analysis. This may indicate that not only the usage of GC, but also the length and cumulative dose of GC matter in the pathogenesis of AVN in SLE [19]. In this study, serositis, PAH, and arthritis were risk factors for AVN in univariate Cox analysis while only arthritis was an independent predictor for AVN in multivariate Cox analysis, which was in consistent with Gladman’s results showing that among the clinical features of SLE, only arthritis was significantly associated with the development of AVN [14]. Probably because previous arthritis may increase the risk of further osteonecrosis by elevating intra-articular hydrostatic pressure, thus causing thrombosis of the epiphyseal vessels. However, other studies found no significant association between arthritis and AVN development [4, 20]. Vascular endothelial injury plays an important role in the pathogenesis of SLE end-organ damages. PAH is a critical manifestation related to vascular deterioration in SLE. Many features of PAH are the result of endothelial cell signal transduction dysfunction, including pulmonary vascular tone, inflammation, pulmonary vessel wall proliferation, remodeling, and in situ thrombosis [21, 22]*.* Present study showed that PAH was a risk factor for AVN in univariate Cox analysis, which may indicate that AVN is derived from abnormal immune response of vasculatures in SLE. However, PAH was not an independent predictor in multivariate Cox analysis, and the correlation between PAH and AVN has not been reported so far. This issue needs further study. NPSLE, renal involvement, vasculitis, serositis, and hematological involvement were related to AVN occurrence in SLE patients in different studies [8, 23–25]. Although various studies explored the associations between organ/system involvement and AVN in SLE patients, conflicting results existed.

SDI is a single validated tool for assessing organ damages [2], which items represent irreversible damages that have occurred after the diagnosis of SLE. Many studies have evaluated the impact of individual organ involvement on future overall or organ-specific damage and have confirmed that patients with pre-existing damage are more prone to develop severer damage in the future [26]. According to our results, multivariate Cox regression analysis suggested that existing organ damage (SDI≥1) at registration was an independent risk factor for AVN development in SLE patients. Probably because this group of patients shared severer disease condition with higher possibility of a higher dose GC administration. This issue has not been investigated in previous studies and deserves further attention.

Despite many studies investigating risk factors for AVN in SLE, few have reported the contribution of specific autoantibodies. Anti-RNP antibodies were detected in 25–47% of patients with SLE and almost all mixed connective tissue disease (MCTD) patients [27]. How the presence of anti-RNP autoantibodies contributes to the pathogenesis of SLE and MCTD, and what clinical symptoms occur as a result of the anti-RNP response remain unclear. This study revealed anti-RNP positivity as an independent predictor for AVN, which was in accordance with the result of a newly published study that positive independent anti-RNP antibody (OR 3.35, 95%CI 0.80–10.73) was significantly associated with osteonecrosis in SLE [28]. Further researches are needed for the exploration of the mechanisms. It has been reported that antiphospholipid antibodies (aPLs) are closely related to thrombus formation while AVN is a comprehensive result of metabolic and local factors affecting blood supply, which may indicate potential role of aPLs positivity in the occurrence of AVN. However, the correlation between aPLs and AVN in SLE remains controversial in previous research [29]. This study did not find an association between aPLs positivity and AVN occurrence. Further explorations are needed to investigate whether positive aPLs could increase the risk for AVN and the underlying mechanisms.

This is the first multi-center cohort study to investigate the predictors for AVN in Chinese SLE patients and found early disease onset of SLE, arthritis, existing organ damage (SDI≥1) at registration, positive anti-RNP, and high maximum daily dose of glucocorticoids as independent risk factors, and developed a risk scoring system, which can help stratify patients at high risk for AVN and facilitate clinical decision making for disease surveillance and intervention. However, limitations exist as the time of AVN occurrence was identified as the time when patients were diagnosed as AVN clinically, mostly symptomatic, thus might underestimate the prevalence of AVN and lacking information of AVN at early stages. Therefore, further research with prospective designing and regular monitoring via MRI may be helpful to provide more information. The study first developed a risk scoring system for the AVN occurrence in SLE patients, which may facilitate clinicians’ quick stratification and identification of patients with higher risk for AVN in future follow-ups, and thus personalize monitoring and early intervention. Since the AUC was not ideal, this preliminary model deserves future study to involve more participants and more comprehensive follow-ups to improve its performance. Another limitation was that the potential influence of other drugs, such as anticoagulants, cholesterol-lowering medications, and bisphosphonates, was not assessed due to a lack of information. In addition, the reason why some patients with the same risk factors (such as glucocorticoid usage) did not develop osteonecrosis remained unclear. This may be because the dysregulated immune micro-environment and the complicated pathogenesis of SLE play important roles in the development of AVN synergistically. For instance, the imbalance between vascular endothelial growth factor (VEGF) and its soluble receptors in SLE may be key triggers for the loss of VEGF expression in osteoprogenitor differentiation into mature bone-forming osteoblasts, which may add to risk of AVN in SLE patients [30].

## Conclusion

In summary, AVN is one common organ damage in SLE. This study demonstrated that early SLE onset, arthritis, existing organ damage (SDI≥1) at registration, anti-RNP positivity, and high maximum glucocorticoid daily dose contributed to the development of AVN independently, thus providing information for early risk stratification of AVN in SLE patients. Physicians should pay more attention to patients with risk factors for AVN and closer monitoring is needed. Further prospective studies with longer follow-ups are required to provide more information on the early identification and prevention of AVN development.

## Data Availability

The datasets used and analyzed during the current study are available from the corresponding author on reasonable request.

## Abbreviations

SLE: Systemic lupus erythematosus
AVN: Avascular necrosis
GC: Glucocorticoid
CSTAR: Chinese SLE Treatment and Research Group
ACR: American College of Rheumatology
SLICC: Systemic Lupus International Collaborating Clinics
PUMCH: Peking Union Medical College Hospital
ANA: Antinuclear antibody
anti-dsDNA: Anti-double stranded DNA antibody
anti-Sm: Anti-Smith antibody
anti-RNP: Anti-ribonucleoprotein antibody
anti-SSA: Anti-Sjogren Syndrome A antibody
anti-SSB: Anti-Sjogren Syndrome B antibody
anti-rib P: Anti-ribosomal P protein antibody
aCL: Anticardiolipin antibody
anti-β2GP1: Anti-β2 glycoprotein 1 antibody
LA: Lupus anticoagulant
SLEDAI-2K: Systemic Lupus Erythematosus Disease Activity Index 2000
SE: Standardized error
IQR: Interquartile range
ROC curve: Receiver operating characteristic curve
AUC: Area under the receiver operating characteristic curve
PAH: Pulmonary arterial hypertension
SDI: Systemic Lupus International Collaborating Clinics/American College of Rheumatology Damage Index
MRI: Magnetic resonance imaging
MCTD: Mixed connective tissue disease
